# Characterizing Federally Mandated Early Intervention for Children with Social Communication Delays: A Mixed-Methods Analysis

**DOI:** 10.3390/bs15030293

**Published:** 2025-03-02

**Authors:** Yael G. Dai, Kyle M. Frost, Ellie M. Harrington, Yael Stern, Emily R. Britsch, Brooke R. Ingersoll, Allison Wainer, Wendy L. Stone, Sarabeth Broder-Fingert, Alice S. Carter

**Affiliations:** 1 Department of Psychology, Florida International University, Miami, FL 33199, USA; 2 Department of Pediatrics, University of Massachusetts Chan Medical School, Worcester, MA 01605, USA; kyle.frost1@umassmed.edu (K.M.F.); sarabeth.broderfingert1@umassmed.edu (S.B.-F.); 3 Department of Psychology, Michigan State University, East Lansing, MI 48824, USA; harr1798@msu.edu (E.M.H.); ingers19@msu.edu (B.R.I.); 4 Rush University Medical Center, Chicago, IL 60612, USA; yael_s_stern@rush.edu (Y.S.); allison_wainer@rush.edu (A.W.); 5 Department of Psychology, University of Washington, Seattle, WA 98195, USAstonew@uw.edu (W.L.S.); 6 Department of Psychology, University of Massachusetts Boston, Boston, MA 02125, USA; alices.carter@umb.edu

**Keywords:** social communication delays, autism, Part C, early intervention

## Abstract

The most common way for children with social communication delays to receive intervention before age three in the United States is through Part C early intervention (EI). Part C was designed to take a multidisciplinary approach to address a range of developmental domains. The type of intervention delivered in Part C EI has rarely been examined through direct observation. Our team conducted a mixed-methods analysis to characterize EI sessions by 33 providers across four states. Specifically, we describe the quantity and quality of caregiver coaching based on provider report and researcher coding of EI session content. Eligible providers conducted weekly EI sessions with at least one child with social communication delays. Providers self-reported greater use of caregiver coaching relative to the video coding conducted by researchers. While there were similarities in session topics, presumed goals, and intervention strategies used across providers, differences were observed in session duration, session location, and caregiver engagement in session. This study begins to fill a substantial gap by illuminating the types of interventions children with social communication delays receive in federally mandated Part C. It also highlights the need for more specialized training and standardization in EI practices to ensure that children with social communication delays and their caregivers benefit from the most efficacious interventions during a critical time of increased brain plasticity.

## 1. Introduction

As part of the Individuals with Disabilities Education Act (IDEA), all children with developmental delays or disabilities in the United States qualify for intervention (Centers for Disease Control and Prevention, 2022; Rosenberg et al., 2013). Part C early intervention (EI) is a federal multidisciplinary interagency system that provides intervention to infants and toddlers from birth to 36 months of age (Schwartz & Sandall, 2010). Part C EI serves approximately four percent of children in the U.S. who are younger than three years old (Early Childhood Technical Assistance Center, n.d.).

Children who receive an autism diagnosis prior to age three are routinely referred to Part C EI in addition to autism-specific services, where available. Estimates suggest that at least 10% of children served by Part C EI are autistic (Eisenhower et al., 2021). However, since Part C EI serves children from birth to three years old with developmental delays, and most children in the United States are not diagnosed with autism until age four (Maenner et al., 2023), it is likely that many children in EI will go on to receive an autism diagnosis after aging out of Part C. Taken together, Part C is the most common way for young children with early indicators of autism to receive EI (Pellecchia et al., 2023).

Given the importance of EI in improving social skills, communication, cognitive functioning, and adaptive behavior in children showing early signs of autism (e.g., social communication delays) and those diagnosed with autism^1^ (Estes et al., 2015; Green et al., 2017; Guthrie et al., 2023; Landa, 2018; Whitehouse et al., 2021), Part C EI offers a powerful opportunity to provide services to these children during a time of critical brain plasticity (Dawson, 2008). Additionally, the delivery of evidence-based intervention through Part C for children with social communication delays can reduce health disparities that arise from ethnoculturally minoritized children being diagnosed with autism later (Angell et al., 2018; Durkin et al., 2015; Liptak et al., 2008; Maenner et al., 2021; Thomas et al., 2007).

Part C specifies a few minimum standards that must be included in EI services. For example, Part C requires that every child receive an evaluation and a service plan that reflects goals that were determined in collaboration with the family (IDEA, 2004; Pickard et al., 2023). Part C EI is also meant to use family-centered care during intervention sessions by involving family members in sessions and valuing their choices (Pickard et al., 2023). However, Part C was designed to take a multidisciplinary approach to addressing a wide range of developmental concerns in children. Therefore, the type of intervention that children with social communication delays receive may vary based on the expertise of the provider with whom they are paired. It is unclear whether the variation in the type of provider delivering services, the content covered in sessions, and the strategies used are optimally suited to support skill development in children with social communication delays. Indeed, most children with social communication delays receive low-intensity, generic interventions rather than autism-specific evidence-based interventions (Aranbarri et al., 2021; Monteiro et al., 2016). Even when children have been diagnosed with autism, services continue to vary widely because few states have guidelines regarding the type and intensity of Part C EI services that autistic children should receive (Aranbarri et al., 2021). Finally, a recent study found that many EI providers endorsed needing more training in autism (70%), in improving caregiver engagement in session (56%), and in caregiver coaching strategies and methods (51%) (Aranbarri et al., 2021). Relatedly, a meta-analysis documented improved outcomes for children with autism who received hospital or university-based intervention versus community intervention (Nahmias et al., 2019). Together, these studies suggest that evidence-based treatments may not be consistently implemented in community settings, and they highlight the need to better understand the characteristics of providers delivering services and what they are doing during Part C sessions with children with social communication delays (Aranbarri et al., 2021; Nahmias et al., 2019).

To date, it is unclear what type of services children with social communication delays receive in EI through Part C. In one of the few examinations of EI for children with social communication delays, Stahmer (2007) found extreme variability in the structure of sessions and the use of interventions considered to be efficacious for autistic youth. However, this study relied on provider interviews. Given the strong empirical support for naturalistic developmental behavioral interventions (NDBIs) for children with social communication delays (Gosling et al., 2022; Schreibman et al., 2015; Song et al., 2024), a recent study compared EI provider reports and the observed use of NDBI strategies (e.g., arranging the environment, following the child’s lead, balancing turns, prompting language and actions, and expanding the child’s focus; Tomeny et al., 2025). They found that early interventionists reported higher-quality practices than what was observed (Tomeny et al., 2025).

Most research characterizing EI sessions has focused on caregiver coaching during sessions (Douglas et al., 2020; Pellecchia et al., 2023; Pickard et al., 2023). Despite increasing consensus about the importance of caregiver coaching for children with social communication delays (Sone et al., 2021) and provider reports that they are frequently coaching caregivers during EI sessions (Aranbarri et al., 2021), the few studies that have coded sessions or asked caregivers about their experiences report low rates of provider coaching during EI sessions through Part C (Aranbarri et al., 2021; Lee et al., 2022; Pellecchia et al., 2023). Additionally, there is tremendous ambiguity and variation in the way parent coaching is defined. For example, a recent meta-analysis noted that coaching is frequently used as a broad term to describe the process of providers giving instruction to caregivers without consensus on the components involved in coaching (Sone et al., 2021).

The current study begins to fill a substantial gap in the literature by characterizing EI Part C sessions for children with social communication delays across four states in the U.S. This contribution addresses crucial gaps in research on multiple fronts. First, considering that EI is frequently the initial point of support for children with social communication delays (whether diagnosed with autism or not), understanding the nature of interventions delivered during this critical period of brain plasticity is paramount. Second, from a research perspective, given that EI often serves as the comparison or “treatment as usual” group in clinical trials that evaluate the effectiveness of novel interventions, characterizing the intervention methods employed by these comparison groups is essential for the interpretation of the results of clinical trials. Third, given an increased focus on dissemination and implementation research, it is important to understand the landscape of usual care and how current practices may influence the adoption and sustainment of new intervention practices. Accordingly, this study aimed to (1) describe the extent of caregiver coaching during session based on (a) provider report and (b) coding of sessions by researchers and (2) characterize the content of EI sessions with children with social communication delays and their caregivers. Based on the limited research in this area, we hypothesized that providers would endorse coaching caregivers more often than coaching strategies would be observed by researchers. Our second aim was exploratory since there is no existing literature to guide the hypotheses. However, due to our sampling strategy, we expected that the majority of sessions across providers would be devoted to building social and communication skills for children.

## 2. Materials and Methods

### 2.1. Participants

This study was part of a larger multi-site hybrid effectiveness–implementation clinical trial examining a type of caregiver-implemented naturalistic developmental behavioral intervention (NDBI), reciprocal imitation teaching (RIT) (Ingersoll, 2012), for toddlers with social communication delays enrolled in Part C EI (NCT–05114538). Part C EI providers in the larger study were recruited from four geographically distributed regions across the United States (Illinois, Massachusetts, Michigan, and Washington) and enrolled if they met the following inclusion criteria: (a) English-speaking, (b) treating at least two children with social communication delays, suspected autism, or diagnosed autism, (c) no prior training in Reciprocal Imitation Teaching (RIT) or another NDBI, (d) willing to receive training in caregiver-implemented RIT, (e) willing to be videotaped during EI sessions, and (f) willing to share the study’s ‘permission to contact’ form and/or give a study handout to all families on their caseload who may be eligible for the study. Toddlers with social communication delays and their caregivers were recruited from enrolled providers’ caseloads if (a) the child had a diagnosis of autism or displayed early social communication challenges, (b) the child was between 16 and 33 months, (c) the child did not have any significant motor, vision, or hearing impairments, (d) the child received at least one weekly session with the participating provider (not co-treated with another provider), (e) the caregiver was present during the EI sessions, (f) the caregiver was the biological parent or custodian guardian, (g) the caregiver was at least 18 years old, and (h) the caregiver spoke English or Spanish. Providers were randomized to either receive training in caregiver-implemented RIT (Ingersoll, 2012) or waitlist–control, in which they continued to deliver treatment as usual. For the current study, only the providers randomized to the treatment-as-usual condition (i.e., standard Part C) who enrolled at least one family into the larger study by January 2024 (*n* = 33) were included in the present study.

Providers in this sample were 26–61 years old (*M* = 40.21, *SD* = 10.69). The majority of providers (*n* = 32) identified as female, and one identified as male. Providers ranged significantly in the number of years of experience in their profession (1 to 40 years; *M* = 11.21 years; *SD* = 9.22 years) and in their experience working with children with social communication delays, including autism (1 to 40 years, *M* = 10.78, *SD* = 8.88). Most providers were speech–language pathologists (39%) or occupational therapists (24%), and the majority of them had a Master’s degree (76%). Additional information about provider demographic characteristics and professional backgrounds is presented in Table 1.

Children in the sample were 18–33 months old (*M* = 26, *SD* = 3.91 months). The majority of the children were male (*n* = 23; 70%). At enrollment, only one child in the sample had a community diagnosis of autism. However, by the end of the 9-month larger study, 27 children (82%) had a formal diagnosis of autism, 4 children (12%) were assessed and autism was ruled out, and 2 children (6%) did not have an autism diagnosis but were lost to follow-up by the study visits during which a diagnostic assessment would have taken place.

The primary caregivers in the sample were 22–55 years old (*M* = 35.58, *SD* = 7 yr). Most of the caregivers identified as female (*n* = 31; 94%). The majority of caregivers did not have a college degree (58% earned a high school diploma or GED, attended trade school or vocational school, received an associate degree, or completed courses towards college without earning a college degree, while 42% earned a college degree, a master’s degree, or a professional degree).

### 2.2. Procedures

As part of the larger study, providers completed three sets of surveys across a 12-month period. Providers were also asked to record three of their EI sessions with each participating family. A researcher assisted with collecting these recordings via a HIPAA-compliant Zoom. For the present study, baseline surveys from the larger clinical trial were used, as this was when provider demographics, professional background, and coaching practices were collected (aim 1a). Additionally, one of the three recorded EI sessions per provider was randomly selected for video coding (aims 1b and 2; coding process described below). Providers were not aware of which of their three recorded sessions was selected for video coding. The duration of observed sessions varied from 22 to 68 min (*M* = 44 min; *SD* = 11 min). Most EI sessions were conducted in person in the family’s home. Six (18%) of the observed sessions were telehealth sessions. Telehealth sessions have become increasingly common in Part C since the start of the COVID-19 pandemic. Goals and strategies are intended to be consistent across telehealth and in-person sessions. However, during telehealth sessions, providers are unable to interact directly with young children; instead, they interact with parents remotely while the parent is with their child. To reflect the breadth of practices in Part C, both in-person and telehealth sessions are included in the analyses.

### 2.3. Measures

***Provider Practices Survey:*** As part of the larger study, providers completed a survey at baseline to report their demographic characteristics and provide information about their typical practices. For the present study, provider race, ethnicity, degree, and training background were reviewed (see Table 1). Providers also reported how often they used NDBI strategies (i.e., labeling a child’s actions, imitating a child’s play or vocalizations, modeling, praising, and prompting) when working with children with social communication delays in the last month (ratings were on a 5-point scale, “Never” to “Always”; see Table 2 for items). Similarly, providers self-reported how often they used coaching strategies (e.g., discussing how to use a strategy, demonstrating, role play, observation, in vivo feedback, planning, problem-solving, and helping the caregiver plan for homework practice; see Table 3 for items). Internal consistency, as measured by Cronbach’s alphas for the 9 NDBI items and the 10 coaching items, was 0.71 and 0.79, respectively.

***Observed Caregiver Coaching Quality:*** The Parent Empowerment and Coaching in Early Intervention (PEACE) caregiver coaching fidelity tool (Pellecchia et al., 2023) measures the quality and use of evidence-based caregiver coaching strategies during EI sessions. The measure was designed to be used with any caregiver-mediated intervention and to measure a provider’s use of coaching without assessing the provider’s use of child-directed interventions (Pellecchia et al., 2024a). PEACE consists of 25 items that are scored on a 5-point scale (“Never Observed” to “Almost Always Observed”). Ratings of 4 or 5 are considered acceptable fidelity of caregiver coaching (Pellecchia et al., 2024a).

The present study used an adapted version of the PEACE coding tool, which included 21 items on the same 5-point scale (see Supplementary Materials). Specifically, the Daily Routines domain was removed, and two questions in the General domain were replaced to reflect the fact that some EI sessions occurred in person, while others took place remotely. The adapted version captures four coaching domains, with each domain including between three and five items: (1) collaboration, which assesses the degree to which the provider cooperates with the caregiver to set goals and make decisions about sessions; (2) demonstration, which captures providers’ explicit teaching and showing of intervention strategies; (3) in vivo feedback, which reflects whether providers observe caregiver–child interactions and comment on their implementation of strategies; and (4) reflection and problem-solving, which assesses providers’ listening to the caregiver, reflecting the caregivers’ statements, checking in about practice, and problem-solving barriers to practicing intervention strategies between sessions. Additionally, a general domain captured the providers’ use of five strategies that are considered good practice to structure sessions and facilitate generalizability (i.e., checking in with the caregiver about updates since the last session, setting an agenda with the caregiver, arranging the environment to promote caregiver–child interaction, maintaining a position that will not interfere with the caregiver–child interaction, and interacting with the child and caregiver together as a dyad). In the general domain, only 3 items were rated for telehealth sessions since 2 of the items were not applicable (e.g., maintaining a position that would not interfere with the caregiver–child interaction). Together, these are considered evidence-based coaching practices, regardless of provider discipline (Pellecchia et al., 2023).

Importantly, while most coaching strategies (e.g., demonstration) are captured by both the provider questionnaire and PEACE, the specific wording of items varied and, therefore, will lead to subtle differences in the construct being captured. For example, PEACE includes 3 questions about demonstration (explicitly teach a strategy to the caregiver, explain the purposes of the techniques implemented, and model techniques that promote caregiver–child interaction; see Supplementary Materials), while the provider survey only included one broader item about demonstration (demonstrate how to use a strategy; see Table 2). Additionally, provider surveys captured frequency, while PEACE ratings reflect both frequency and quality, such that low scores may indicate the absence of a strategy or incorrect use of a strategy. Item-level scores are averaged to produce domain scores (see Supplementary Materials). Research assistants were trained to a threshold of 80% on three consecutive videos prior to coding. A random sample of 20% of the videos were double-coded for reliability. A two-way random intraclass correlation (range = 0.57–0.86) indicated moderate-to-good reliability between the two coders across domains.

***Qualitative Analysis of Session Content:*** An adapted Rapid Qualitative Analysis approach (Hamilton & Finley, 2019; Nevedal et al., 2021; Vindrola-Padros & Johnson, 2020) was used to summarize the content of EI sessions, including both child-directed intervention strategies and interactions between therapists and caregivers. To develop a rapid analysis strategy and template, five members of the research team first watched segments of three EI session videos to (a) discuss the planned focus of the qualitative analysis and (b) develop a summary template that could capture the focus areas of interest. The research team engaged in dialog, discussing different perspectives until a consensus was reached. The final summary template included the following sections: (1) extent and description of caregiver involvement; (2) primary child-focused intervention strategies discussed or observed; (3) session goals; (4) topic areas discussed in session; and (5) general impressions about session quality and rapport. Summaries comprised a few sentences of text for each section of the template. Although our team had no a priori codes, given our expertise and the broader study context, we were particularly attuned to noting the presence/absence of caregiver coaching and NDBI strategies. Each member of the team then applied the template to independently summarize the same three videos to calibrate the level of detail included in summaries and to confirm that similar content was captured across team members. The remaining 30 videos selected for summarizing were then randomly divided among raters and summarized independently.

### 2.4. Analytic Strategy

***Quantitative Analysis:*** Descriptive statistics were used to examine provider demographics, education, professional background, and self-reported practices about the frequency of use of strategies during typical EI sessions with children with social communication delays. Descriptive statistics were used to examine means and standard deviations across providers. Additionally, we used a repeated measures analysis of variance (ANOVA) to examine differences between PEACE domain scores. Paired sample *t*-tests were used for follow-up comparisons.

***Qualitative Analysis of Session Content and Child-Focused Strategies:*** A team of clinical researchers with expertise in autism intervention charted the qualitative summaries of session content in a data matrix (respondent*domain) so data could easily be reviewed for similarities, differences, and patterns across sessions (Averill, 2002). Five members of the research team independently reviewed the data matrix and developed preliminary themes describing the EI sessions. The group then met twice (for approximately 4 h total) to discuss and develop a final set of themes and subthemes that summarized EI session content.

## 3. Results

***Provider Report of Practices.*** When describing their typical practices in working with children with social communication delays, providers reported a wide range of variation in the percent of time they spend working directly with the child (10 to 90% of sessions; *M* = 35%, *SD* = 21%) and in the percentage of time that they allocate to coaching caregivers during a typical session (4 to 70% of sessions, *M* = 32%, *SD* = 15%). Overall, providers reported frequently using caregiver coaching during EI sessions with children who had social communication delays. Specifically, the majority of providers reported that they often or always discuss their use of strategies with caregivers (*M* = 4.45, *SD* = 0.51), demonstrate how to use a strategy (*M* = 4.24, *SD* = 0.50), observe the caregiver practicing using strategies with their children (*M* = 3.94, *SD* = 0.75), provide feedback to caregivers on their use of strategies (*M* = 4.03, *SD* = 0.73), help caregivers plan for practice between sessions (*M* = 3.96, *SD* = 1.02), review the caregivers’ experience of practicing between sessions (*M* = 3.84, *SD* = 0.91), and help caregivers problem-solve challenges in practicing and implementing strategies (*M* = 4.15, *SD* = 0.71; see Table 2). In contrast, providers reported that they rarely show caregivers videos (*M* = 2.0, *SD* = 1.0), role-play with caregivers (*M* = 2.82, *SD* = 0.95), or use a sequenced curriculum to teach the caregiver a set of strategies during EI sessions (*M* = 2.15, *SD* = 1.03).

When working directly with children with social communication delays, most providers reported that they often or always label children’s actions (*M* = 4.27, *SD* = 0.57), imitate the child’s vocalizations (*M* = 4.24, *SD* = 0.56), model new play actions (*M* = 4.12, *SD* = 0.74) and gestures (*M* = 4.12, *SD* = 0.74), and praise children for imitating them (*M* = 4.42, *SD* = 0.71). Strategies that providers endorsed less frequently included imitating children’s play (*M* = 3.94, *SD* = 0.93) and movement (*M* = 3.94, *SD* = 0.93), physically prompting children who do not imitate (*M* = 3.79 *SD* = 0.74), and praising children after physically prompting them to use a new action (*M* = 3.97, *SD* = 0.1.07; see Table 3).

***Observed Use of Caregiver Coaching.*** In contrast to providers’ reports, coders rated providers as using fewer coaching strategies in the observed sessions (see Table 4). Specifically, providers inconsistently implemented general strategies to facilitate coaching in session, such as setting a plan with the caregiver or arranging the environment to promote caregiver–child interaction.

Across all collaboration items, providers were rated as infrequently collaborating with caregivers. Specifically, most providers never or rarely collaborated with caregivers in setting goals (*M* = 1.45, *SD* = 0.87), letting caregivers make some decisions and lead parts of the intervention session (*M* = 2.18, *SD* = 1.10), using and expanding upon caregiver ideas during session (*M* = 1.54, *SD* = 1.12), or asking for caregiver input or inviting feedback on their observations (*M* = 1.42, *SD* = 0.90). Similarly, providers rarely used strategies from the demonstration domain, including explicitly teaching a strategy to the caregiver (*M* = 1.33, *SD* = 0.65), explaining the purpose of the techniques implemented (*M* = 1.61, *SD* = 0.99), or demonstrating/modeling techniques that promote caregiver–child interaction (*M* = 1.33, *SD* = 0.60). In addition, most providers never or rarely provided in vivo feedback, such as allowing sufficient time for the caregiver to practice strategies during session (*M* = 1.88, *SD* = 1.29), providing positive feedback based on observation of caregiver–child interactions in session (*M* = 1.67, *SD* = 1.05), and providing constructive feedback about caregiver actions during session (*M* = 1.82, *SD* = 1.31). In contrast, the reflection and problem-solving domain had higher rates of utilization (*M* = 3.06, *SD* = 0.75). Still, there was marked variation in the frequency of behaviors across this domain. Specifically, providers often or almost always answered caregiver concerns when caregivers raised concerns (*M* = 4.81, *SD* = 0.54), listened to what the caregivers had to say (*M* = 4.72, *SD* = 0.80), and helped caregivers work through obstacles in implementing the techniques during sessions using reflective strategies (*M* = 5, *SD* = 0). Of note, these three items were the only PEACE items that included a “No Opportunity” option as part of the coding system. Providers sometimes asked caregivers questions about routines, the use of strategies, or the child’s actions (*M* = 3.21, *SD* = 1.34) and rarely discussed progress with the caregiver (*M* = 2.36, *SD* = 1.25) or asked the caregiver about possible barriers to practice and discussed solutions (*M* = 1.36, *SD* = 1.03).

A repeated measures ANOVA was conducted to assess differences across the domains of the PEACE scale. The results revealed a significant difference among PEACE domains, *F* (3,30) = 43.23, *p* < 0.001, η^2^ = 0.81. Post hoc comparisons showed that the reflection and problem-solving domain was rated as significantly higher than all other domains (see Table 4). No other pairwise comparisons were significant (see Table 4).

Although the Provider Report of Practices survey and the PEACE coding did not contain identical questions, preventing a direct one-to-one item comparison, both measures capture important aspects of provider practices related to caregiver coaching. Table 5 summarizes similar items from the measure in a side-by-side comparison.

***Qualitative Analysis of Session Content.*** The research team developed two major themes and several subthemes via the Rapid Qualitative Analysis of 33 session videos (one per provider). The first theme, “common practices”, was reflective of practices that were present across most EI sessions regardless of provider training background or family characteristics and included four subthemes: (a) loose session structure and focus, (b) intervention strategies used with children, (c) session topics, and (d) emphasis on rapport. The second theme, “variability in caregiver coaching and engagement”, was reflective of the wide variety of ways in which EI providers engaged caregivers.

### 3.1. Theme 1. Common Practices

Despite differences in the characteristics of children, families, EI providers, and EI systems, there were several features that were observed with a high degree of consistency across the observed sessions. This included a loose session structure/focus, similarities in the types of intervention strategies used, similar session topics, and an emphasis on rapport. Each subtheme is described in more detail below.

***Subtheme 1a: Loose Session Structure and Focus.*** Observed EI sessions tended to unfold organically without an explicit plan, goal, or focus identified during the session. Although intervention goals were presumably set in collaboration with the families prior to beginning EI, the coding team did not observe explicit discussion or review of goals in session. Session goals were often unclear to the expert observers and could only be inferred by the types of child-directed strategies that providers were observed to implement. Providers often checked in with caregivers about updates on children’s behavior and skills (e.g., whether the child was using any new words) but did not check in about whether caregivers practiced any intervention strategies or how the practice had gone. Sessions tended to conclude without any kind of clear closing activity, wrap-up, or plan for the upcoming week.

***Subtheme 1b: Child-Focused Intervention Strategies.*** Almost all sessions included frequent use of child-led play, speech with excited vocal tone and sound effects directed to the child, imitation of the child’s vocalizations, labeling (i.e., naming toys the child is using or looking at, naming a toy a provider picks up, and describing a toy or child’s actions), and modeling play. In some sessions, providers were also observed to imitate the child’s actions. Finally, providers often prompted children to demonstrate a skill and provided praise as reinforcement. While these behavioral strategies were frequently observed, the quality and effectiveness of these behavioral strategies was generally limited. For example, therapists often cued children to say or do something but did not provide support or follow-through (e.g., repeated prompts, scaffolding) if the child did not respond. Similarly, providers maintained a positive attitude and frequently praised children, but praise was general rather than specific, and positive reinforcement (e.g., praise, giving a desired object) was often not contingent upon the children’s actions. Lastly, the pacing of behavioral teaching strategies or demands placed was not consistently adjusted to the child’s level of engagement. At times, this led to child frustration or disengagement from sessions.

***Subtheme 1c: Session Topics.*** Although goals were not stated explicitly during sessions, most sessions appeared to focus on promoting children’s verbal and non-verbal communication, as indicated by the provider’s frequent use of labeling and signing and their questions to caregivers about the children’s progression in using language. Other discussion topics during session included checking in about children’s adaptive skills (e.g., toilet training, clean-up), development (motor, play, toileting, eating, sleep, sensory regulation, behavioral challenges), or service coordination (Individualized Education Program Meetings, assessment, school transitions, preschool). When these topics were discussed, providers tended to ask about updates, validate the caregiver’s experience, and sometimes provide suggestions. However, such discussions were observed infrequently and lasted no more than a few minutes, taking up a small percentage of session time.

***Subtheme 1d: Emphasis on Rapport.*** In most of the observed EI sessions, providers appeared to have established a positive rapport with caregivers, as evidenced by shared positive affect, positive interactions, and caregiver involvement in the session, speaking to EI providers’ skills in providing family-centered care. In the rare instances in which poor rapport was noted, providers were more directive, solicited minimal input from caregivers, or appeared to lack shared goals with the caregiver, as evidenced by the caregiver’s disagreement with the providers’ statements or minimizing providers’ recommendations.

### 3.2. Theme 2. Variability in Caregiver Coaching and Engagement

The caregivers’ roles in sessions were highly variable, ranging from passive observation to intermittent engagement to involvement in all aspects of the session. For example, in some sessions, caregivers were observed to be nearby but engaged in their own activity (e.g., knitting, on a computer) while the provider interacted with the child. In contrast, some sessions appeared to be more caregiver-led, with the caregiver structuring activities and interacting with the child the most, with occasional direction or participation by the provider. Commonly, the provider, caregiver, and child all interacted together, with frequent, fluid shifts between primary interaction partners (e.g., caregiver plays with the child, provider comments on the play and asks a question, provider and caregiver have a brief conversation, then the provider plays with the child for a few minutes while the caregiver occasionally interjects). Consistent with the loose and fluid session structure described above, the use of structured caregiver coaching strategies was rarely observed. Providers often engaged caregivers by asking reflective questions or offering a brief suggestion by describing something for the caregiver to try. We rarely observed providers explicitly modeling an intervention strategy for the caregiver’s benefit, having the caregiver practice while the provider gave supportive feedback, or helping the caregiver develop a plan for how to use intervention strategies at home.

## 4. Discussion

Prior research has emphasized the importance of understanding the extent to which evidence-based interventions are implemented in publicly funded EI. This is one of the few studies describing both the structure and content of Part C EI services for young children with social communication delays, extending beyond the exploration of caregiver coaching and leveraging data collected across four states.

As shown in Table 5, providers endorsed the frequent use of caregiver coaching practices in sessions, but ratings of these sessions by trained observers using both quantitative measures as well as qualitative analysis revealed limited use of evidence-based coaching strategies. Importantly, PEACE ratings include coaching practices that providers were asked about on self-report measures (e.g., demonstration, in vivo coaching) and also reflect other important aspects of coaching, such as collaboration. Additionally, PEACE includes more items about coaching, thereby capturing a more holistic view of provider coaching practices in EI. Our findings are consistent with previous research showing the infrequent use of caregiver coaching in EI (Lee et al., 2022; Pellecchia et al., 2023) despite providers self-reporting a high use of these strategies (Aranbarri et al., 2021). This finding may reflect a knowledge and training gap with regard to components of evidence-based caregiver coaching. The results of our qualitative analysis provide helpful context for these discrepant findings between provider self-report and observational ratings. A structured coaching approach (e.g., explicit teaching and modeling, in vivo observation and feedback) was rarely observed. Although providers generally did not explicitly teach or model behaviors (e.g., “I’m going to try labeling the toys as we play—Watch how I use simple language to describe what we are doing together”), providers did typically engage in appropriate developmental intervention strategies with children while caregivers were present, which they may interpret as modeling. Similarly, although providers did not generally provide in vivo feedback based on observation of the caregiver using specific intervention strategies in session, providers often did watch caregivers engage with their child—occasionally offering a suggestion or reflective comment—which they may interpret as observation with in vivo feedback.

Importantly, there is a lack of consensus on how caregiver coaching is defined, which may contribute to the discrepancies observed between self-report and observational coding by researchers who specialize in autism intervention. Sone et al. (2021) highlight a range of strategies in caregiver-implemented interventions, distinguishing between caregiver coaching, which involves direct practice in session with feedback, and other caregiver instructional strategies, including reflection and problem-solving. Providers in our study were rated higher on the use of reflection and problem-solving in session but lower in direct practice with in vivo feedback. However, providers in this study self-reported using all of the coaching strategies they were asked about, suggesting that they perceive themselves as engaging in all parts of coaching frequently when they may be engaging in only a subset of coaching practices during many EI sessions. This has important implications for future EI practices because meta-analyses show large positive effects of coaching on caregiver outcomes compared to other instructional strategies (Sone et al., 2021).

Prior research has hypothesized that the strong emphasis on family-centered care in EI serves as a barrier to implementing caregiver coaching because providers must constantly weigh decisions about shifting focus to align with a caregiver’s current needs (Pickard et al., 2023). For example, Pickard et al. (2023) suggested that caregivers’ comments and concerns regarding their children’s behavior or family stressors (e.g., housing eviction) may understandably limit a provider’s ability to implement evidence-based interventions as they were designed. Indeed, providers report several barriers to coaching caregivers, including caregiver-stated preferences that the EI provider works directly with their child while the caregiver manages other family stressors (e.g., Pellecchia et al., 2024b; Tomczuk et al., 2022). However, in the sessions observed for the current study, very little time was allocated to discussing family stressors or topics raised by caregivers, despite the fact that caregivers were present for the session duration. While any given topic (e.g., toilet training) may not have been observed often, given that its relevance varies by child age and developmental level, the fact that little time was allocated to discussing any family topics raised in session suggests that it is unlikely that negotiating family stresses serves as the primary barrier for caregiver coaching. An alternative hypothesis is that providers make the decision not to implement coaching practices due to beliefs about some families simply “not being a good fit” (Tomczuk et al., 2022). Additionally, in prior work, providers reported a need for more training to feel confident in implementing caregiver coaching and in engaging caregivers during sessions (Aranbarri et al., 2021). Our findings that providers use strategies with children and explain these strategies but are less directive in coaching caregivers and providing in vivo feedback may reflect these barriers to implementing caregiver coaching. Additionally, it is possible that providers tend to focus less on coaching caregivers because their attention is commonly and rapidly drawn to their interactions with children. However, since caregiver involvement is a stated focus of Part C intervention, and providers trained in caregiver coaching have demonstrated the ability to integrate coaching caregivers while interacting with children in sessions, this would again point towards the need for increased training in caregiver coaching.

The qualitative analysis of sessions revealed a similar pattern for the use of child-directed strategies. Specifically, when considering both the frequency and quality of strategies (in the qualitative analysis), researchers noted that some strategies were lacking in session despite provider reports indicating the frequent use of these strategies. Providers’ self-reports were consistent with direct observations for the frequent use of labeling children’s objects and actions, imitation of vocalizations and actions, and praise. Additionally, researchers agreed that providers used prompting strategies often. However, direct observation revealed that the quality of behavioral teaching strategies was often limited. For example, when children did not perform an action after prompting, providers did not provide additional support to help children follow through. Therefore, although providers may be using the strategies they endorsed, there may be room for further professional development of evidence-based skills in relation to the frequency, extent, or quality of the use of these strategies. This is perhaps not surprising given that most of the observed providers were either speech–language pathologists or occupational therapists, who may not receive extensive training in behavioral teaching approaches.

Our team was surprised to observe the wide range in duration of sessions and caregiver and child involvement in sessions, indicating that children with social communication challenges appear to be receiving very different intervention experiences in EI, with sessions varying with respect to dosage and the use of evidence-based strategies. However, the content of session topics was largely consistent across providers.

These findings should be interpreted in the context of a few limitations. Most notably, the sample size was small, and providers who choose to participate in a research project may not be representative of the population of EI providers. These factors potentially limit the generalizability of reported findings. Additionally, only one EI session per provider/family dyad was coded. Together, these tapes provide a breadth of topics and strategies used during EI sessions for children with social communication delays. However, one session is not necessarily a reliable measure of provider practices in EI. Relatedly, we did not account for the amount of time that elapsed between the recorded session and when a child began EI. Moreover, although consistent with the demographic characteristics of EI providers, there was very limited racial–ethnic diversity among the EI providers who participated in this study. Lastly, a condition of the study was that caregivers were present for the children’s sessions. Several areas also emerge as interesting future directions. For example, future studies with larger sample sizes can explore ways that in-person EI sessions differ from telehealth sessions with regard to providers’ use of caregiver- and child-directed strategies. Similarly, studies with larger sample sizes could explore the role of provider experience and professional background in provider practices.

The study also has notable strengths. First, the sample included providers who were recruited from multiple EI agencies, as well as both salaried agency-based and contingent workers in the Part C EI system across four states. Therefore, the results better characterize EI across the U.S. rather than only informing practices in a specific region. Second, our mixed-methods approach enabled us to compare self-reported and observed provider practices.

Taken together, these findings have substantial implications for professional development and policy-making, offering insights into refining EI practices. For example, the vast variation observed in sessions suggests that providers and families may benefit from more guidance regarding increasing the duration and structure of sessions. Additionally, some EI providers may benefit from encouragement to explain their own strategy selections and to integrate reflective listening and collaborative goal setting with increased directiveness in coaching. This may foster a deeper understanding of providers’ practices, improve communication with families, and support a more collaborative and transparent approach to intervention, helping families better understand and implement strategies between sessions. Finally, these findings underscore the importance of ongoing professional development opportunities for EI providers, including in the area of autism, as many providers continue to report low confidence in addressing autism-related needs, despite its growing prevalence.

## Figures and Tables

**Table 1:** Provider demographics and professional background.

**Professional Background**	**N (%)**
Speech–Language Pathologist	13 (39.4)
Occupational Therapist	8 (24.1)
Developmental Therapist	4 (12.1)
Early Childhood Educator	2 (6.1)
Special Educator	2 (6.1)
Social Worker	2 (6.1)
Other	2 (6.1)
**Degree**	
Doctoral	2 (6.1)
Master’s	25 (75.7)
Bachelor’s	5 (15.2)
Other	1 (3)
**Race**	
White	25 (75.8)
Asian	2 (6.1)
Black	2 (6.1)
More than one race	4 (12)
**Ethnicity**	
Hispanic/Latinx	1 (3)
Not Hispanic/Latinx	31 (94)
Prefer not to answer	1 (3)

**Table 2:** Provider-reported use of strategies with children with social communication delays.

Strategy (N%)	Always	Often	Sometimes	Rarely	Never
Label child’s actions	11 (33.3)	20 (60.6)	2 (6.1)	0	0
Imitate child’s play	9 (27.3)	16 (48.5)	6 (18.2)	1 (3)	1 (3)
Imitate child’s vocalizations	10 (30.3)	21 (63.6)	2 (6.1)	0	0
Model new play action or gesture for child	10 (30.3)	18 (54.5)	4 (12.1)	1 (3)	0
Praise child for imitating	17 (51.5)	14 (42.5)	1 (3)	1 (3)	0
Prompt child	5 (15.2)	17 (51.5)	10 (30.3)	1 (3)	0

**Table 3:** Provider-reported frequency of coaching strategies used in EI sessions.

Strategy (N%)	Always	Often	Sometimes	Rarely	Never
Discuss how to use a strategy with a caregiver	15 (45.5)	18 (54.5)	0	0	0
Demonstrate how to use a strategy	9 (27.3)	12 (69.7)	1 (3)	0	0
Show caregiver video example of strategy	1 (3)	1 (3)	7 (21.2)	12 (36.4)	12 (36.4)
Role-play strategy with caregiver	2 (6.1)	5 (15.2)	12 (36.4)	13 (39.4)	1 (3)
Observe caregiver practicing strategy with their child	7 (21.2)	18 (54.5)	7 (21.2)	1 (3)	0
Provide feedback on caregiver’s use of strategy	8 (24.2)	19 (57.6)	5 (15.2)	1 (3)	0
Help caregiver plan for practice between sessions	12 (36.4)	11 (33.3)	8 (24.2)	1 (3)	1 (3)
Discuss the caregiver’s experience of practice between sessions	7 (21.2)	17 (51.5)	7 (21.2)	1 (3)	1 (3)
Help caregiver problem-solve challenges when practicing strategies	11 (33.3)	16 (48.5)	6 (18.2)	0	0
Use a sequenced curriculum to teach the caregiver a set of strategies	1 (3)	1 (3)	10 (30.3)	11 (33.4)	10 (30.3)

**Table 4:** Observational coding of providers’ use of caregiver coaching strategies.

Domain	Range	Mean	SD
General Practices	1–3.67	2.80	0.78
Collaboration	1–3.75	1.65 _a_	0.65
Demonstration	1–3.33	1.42 _a_	0.59
In Vivo Feedback	1–5	1.79 _a_	1.03
Reflection and Problem-Solving	1.66–4.20	3.06 _b_	0.75

Note: The range represents the average of each provider’s domain score. Subscripts with different letters indicate significant pairwise comparisons in a repeated measures analysis of variance (*p* < 0.001).

**Table 5:** Comparison of coaching practices based on provider report and researcher coding.

Provider Report		Researcher Coding	
Item	M (SD)	Item	M (SD)
Discuss their use of strategies with caregivers	4.45 (0.51)	Explain the purpose of the techniques implemented	1.61 (0.99)
Demonstrate how to use a strategy	4.24 (0.50)	Demonstrate/model techniques that promote caregiver–child interaction	1.33 (0.60)
Observe the caregiver practicing using strategies with their children	3.94 (0.75)	Allow sufficient time for the caregiver to practice strategies during session	1.88 (1.29)
Provide feedback to caregivers on their use of strategies	4.03 (0.73)	(A) Observe interactions and comment on specific strategies that are working well (B) Observe interactions and provide constructive feedback about current actions	1.67 (1.05); 1.82 (1.31)
Help caregivers plan for practice between sessions	3.96 (1.02)	Ask caregiver about possible barriers to practice and discuss solutions	1.36 (1.03)
Review caregivers’ experience of practicing between sessions	3.84 (0.91)	Ask caregivers questions about routines, use of strategies, or the child’s actions	3.21 (1.34)
Help caregivers problem-solve challenges to practicing and implementing strategies	4.15 (0.71)	Helped caregivers work through obstacles in implementation of the techniques during session using reflective strategies	5 (0)

Note: Both measures used 5-point rating scales (“Never” to “Always”).

## Data Availability

De-identified data presented in this study are available on request from the corresponding author.

## Note

1 We attempt to use inclusive language with the understanding that there is no consensus about preferred language. Therefore, we included both identify-first and person-first language to maximize inclusivity.

## Supplementary Materials

The following supporting information can be downloaded at: https://www.mdpi.com/article/10.3390/bs15030293/s1.
