# Supplementary material for: Characterizing Federally Mandated Early Intervention for Children with Social Communication Delays: A Mixed-Methods Analysis
Source: Behav Sci (Basel). 2025 Mar 2;15(3):293. doi: 10.3390/bs15030293 (PMC11939554; doi:10.3390/bs15030293)
Supplement: Supplementary file 1 [file behavsci-15-00293-s001.zip › behavsci-3407721-supplementary.pdf]

Perelman School of Medicine
UNIVERSITY of PENNSYLVANIA

PEACE
Parent Empowerment and Coaching in Early Intervention

Rater: **Date:** **Video ID:** **Primary or Reli:**

Caregiver Coaching Fidelity Form PILOT TEST							
When there was an opportunity, did the provider	*Almost Always*	*Often*	*Sometimes*	*Rarely*	*Never*	*No Opportunity*	*Comments*
**General**					**General Mean:**		
1. Check in with caregiver about any updates since last session.	5	-	-	-	1	-	
2. Set the plan for today's session with caregiver.	5	-	-	-	1	-	
3. Arrange aspects of the environment to promote parent-child interaction.	5	4	3	2	1	-	
4. Maintain a position that would not interfere with the parent-child interaction.	5	4	3	2	1	-	
5. Interact with the child and the caregiver together as a dyad, rather than separately.	5	4	3	2	1	-	
**Collaboration**						**Collaboration Mean:**	
6. Let caregivers make some decisions and lead parts of the intervention session.	5	4	3	2	1	-	
7. Use and expand caregiver ideas during session.	5	4	3	2	1	-	
8. The coach and caregiver collaboratively set goals for child.	5	4	3	2	1	-	
9. Ask for caregiver input or invite feedback on what is observed.	5	4	3	2	1	-	
**Demonstration**							**Demonstration Mean:**
10. Explicitly teach a strategy to the caregiver.	5	4	3	2	1	-	
11. Explain the purpose of techniques implemented.	5	4	3	2	1	-	
12. Demonstrate/model techniques that promote caregiver-child interaction.	5	4	3	2	1	-	

Perelman School of Medicine
UNIVERSITY of PENNSYLVANIA

PEACE
Parent Empowerment and Coaching in Early Intervention

When there was an opportunity, did the provider	*Almost Always*	*Often*	*Sometimes*	*Rarely*	*Never*	*No Opportunity*	*Comments*
**In-Vivo Feedback**						**In-Vivo Feedback Mean:**	
13. Observe ongoing interactions and comment on specific strategies that are working well (*positive* feedback).	5	4	3	2	1	-	
14. Observe ongoing interactions and provide (*constructive*) feedback about current actions.	5	4	3	2	1	-	
15. Allow sufficient time for the caregiver to practice strategies during session.	5	4	3	2	1	-	
**Reflection and Problem Solving**						**Ref & Prob Solv Mean:**	
16. Answer caregiver concerns.	5	4	3	2	1	N	
17. Listen to what the caregiver has to say.	5	4	3	2	1	N	
18. Evaluate progress with the caregiver.	5	4	3	2	1	-	
19. Ask caregiver questions about routines, use of strategies, or child's actions.	5	4	3	2	1	-	
20. Helps the caregiver work through any obstacles in the implementation of the techniques using reflective strategies.	5	4	3	2	1	N	
21. The coach asks the caregiver about possible barriers to practice and discusses solutions.	5	4	3	2	1	-	
**Overall Fidelity Score (Mean of all items scored):**							

Length of session recording:
Video Quality (1-Poor through 5-Excellent):

Other Notes:
